# Dual-Mediation Paths Linking Corporate Social Responsibility to Employee’s Job Performance: A Multilevel Approach

**DOI:** 10.3389/fpsyg.2020.612565

**Published:** 2021-01-14

**Authors:** Miaoying Fang, Peng Fan, Surya Nepal, Po-Chien Chang

**Affiliations:** ^1^Department of Human Resource Management, School of Economics and Management, Dongguan University of Technology, Dongguan, China; ^2^Department of International Business and Management, School of Economics and Management, Dongguan University of Technology, Dongguan, China; ^3^Department of Business Administration, Changwon National University, Changwon, South Korea; ^4^Department of Management and Administration, Macau University of Science and Technology, Taipa, Macau

**Keywords:** corporate social responsibility, industrial relations climate, job performance, multilevel approach, psychological contract fulfillment

## Abstract

This study attempts to examine the direct impact of corporate social responsibility (CSR) initiatives on employees’ job performance and the indirect relationships between CSR initiatives on employees’ job performance *via* industrial relations climate and psychological contract fulfillment. Data were collected from 764 supervisor–subordinate dyads and 271 middle managers from 85 companies. Using a multilevel approach, the results showed that organizational-level CSR was positively related to employees’ job performance. Moreover, the industrial relations climate and psychological contract fulfillment played mediating effects between CSR initiatives and job performance. This study provides novel theoretical evidence for why and how CSR initiatives improve job performance. Theoretical and practical implications for implementing CSR initiatives are discussed.

## Introduction

Corporate social responsibility (CSR) refers to “context-specific organizational actions and policies that take into account stakeholders’ expectations and the triple bottom line of economic, social, and environmental performance” (Aguinis, 2011, p. 855). After the global financial crisis in 2008, CSR has received a great deal of attention in the management field of research (Wang et al., 2017; Xiao et al., 2019). As a beneficial source of an organization’s competitiveness and long-term sustainability, scholars have discussed the significant role of CSR at different levels. In the initial stage of CSR studies, researchers mainly focused on the macro perspective, exploring the role of CSR in organizational outcomes, such as financial performance (Godfrey et al., 2009) and organizational performance (Lindgreen et al., 2009). Alternatively, some scholars have concentrated on the association between CSR and individual outcomes at the microlevel (e.g., Lee et al., 2013; De Roeck et al., 2014; Glavas and Kelley, 2014). As a result, research fragmentations have been exacerbated by applying different theoretical frameworks (Aguinis and Glavas, 2012; Glavas, 2016). The understanding of the mechanisms linking CSR with employee outcomes seems to have been overlooked (Wang et al., 2017). In particular, the empirical testing of multiple mediation mechanisms based on individual perspective analysis remains limited (Glavas, 2016). Therefore, a multilevel approach to the mediation mechanism is key to understanding the underlying mechanisms of the relationship between CSR initiatives and employee outcomes.

To bridge the significant gaps of prior studies, this study attempts to provide dual-mediation paths linking CSR initiatives to employees’ job performance with the adoption of a multilevel approach. Based on the social identity theory (SIT) (Tajfel and Turner, 1986) and social information processing theory (SIPT) (Salancik and Pfeffer, 1978), our study explores the direct effects of CSR initiatives on job performance and the indirect effects of CSR initiatives on job performance mediated *via* the industrial relations climate (IRC) construct. Although many of the micro-CSR studies have explored numerous mediating factors (e.g., organizational identification), scholars have suggested that future research should provide meaningful insights into variables related to stakeholder relations (Aguinis and Glavas, 2012). As an important element of organizational culture, the IRC refers to the quality of labor–management relations, and thus, it is likely to influence and be influenced by employee–management interactions in the workplace (Pyman et al., 2010). With rapid changes in the economic environment during the last two decades, industrial relations are considered as key factors capable of addressing CSR issues, with many Chinese enterprises making their best efforts to develop workplace relations aimed at generating outstanding performance (Chan and Nadvi, 2014). Therefore, this study endeavors to examine the significant impact that CSR initiatives mediated *via* IRC has on employee behavioral outcomes.

**FIGURE 1:**
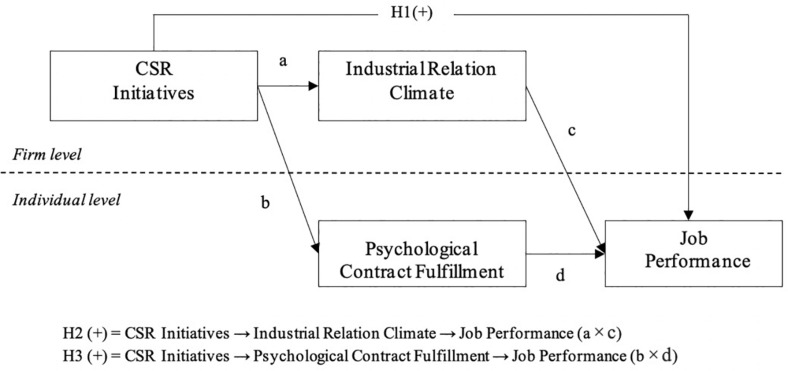
Conceptual model.

Regarding the social exchange theory (SET) (Blau, 1964), we seek to identify whether CSR initiatives may affect job performance through psychological contract fulfillment (PCF). As proposed by initial scholars, a psychological contract is considered to be an important means to understand the relationship between organizations and employees (Rousseau, 2001; Kutaula et al., 2020). It involves emotions, perceptions, and expectations as well as the urges for sensemaking in the workplace (Persson and Wasieleski, 2015). Thus, organizations could, at the best level, address most of the employees’ expectations, thereby contributing to certain aspects that an organization takes care of Dixon-Fowler et al. (2019). Although CSR initiatives are driven by various stakeholders that have a powerful effect on employees’ expectations, most studies have focused mostly on external stakeholders, and the relationships between CSR initiatives and internal stakeholders (i.e., employees) have rarely been investigated (Aguinis and Glavas, 2012; Dixon-Fowler et al., 2013). Therefore, we incorporate PCF into the hypothesized model and explore the mediating mechanism between CSR initiatives and employee job performance (Figure 1).

This study makes several contributions to the literature. First, our study extends CSR research by identifying the mediating mechanism underlying the CSR–employee outcomes relationship. Although CSR scholars have investigated various mediators between CSR initiatives and individuals’ outcomes (e.g., Farooq et al., 2014; Glavas and Kelley, 2014), multiple mediating mechanisms have not received much consideration (Glavas, 2016). This study fills previous gaps by providing a better understanding of CSR impacting on the employees’ behavioral outcomes (i.e., job performance) through the mediating effect of IRC and PCF. Second, this study explores the approach of measuring CSR on multiple levels (Aguinis and Glavas, 2012). Numerous scholars have accessed an organization’s CSR on an individual level and investigated the relationship between perceived CSR and different outcomes, such as job satisfaction (De Roeck et al., 2014), normative organizational commitment (Mory et al., 2016), and socially responsible behaviors (De Roeck and Farooq, 2018). However, multilevel research including both organizational and individual-level data is lacking (Jones et al., 2017). Thus, this study enriches the knowledge of microfoundations applicable to the CSR literature by utilizing a multilevel approach. Third, researches so far have put emphasis on Western contexts and lack enough empirical research related to Asia (Kutaula et al., 2020). The current study also makes an empirical contribution to the CSR literature by examining the role of CSR initiatives in employees’ job performance in the Chinese organizational context.

## Theoretical Background and Hypotheses

### CSR Initiatives and Job Performance

Based on SIT, individuals consider themselves as salient to social categories, and individuals’ self-conception is influenced by relevant social groups or organizations. Consequently, the social identity (ingroup or outgroup) may have a critical influence on people’s attitudes and behaviors (Tajfel and Turner, 1986; Ali et al., 2020). Researchers offer empirical evidence of the vital role of CSR initiatives in affecting individuals’ outcomes. Edwards and Kudret (2017) extended the CSR literature by exploring the influence of multifocal CSR perceptions on in-role job performance *via* affective commitment and organizational pride. Newman et al. (2019) investigated the impact of perceived CSR practices on employees’ job performance and organizational citizenship behavior (OCB), which includes their positive contributions to their work environment that are not formally part of their job descriptions. Furthermore, a recent study demonstrated that external CSR initiatives could improve employees’ job performance, while both external and internal CSR initiatives play indirect roles in improving job performance *via* job satisfaction (Story and Castanheira, 2019).

According to SIT, employees evaluate themselves *via* their organization’s social status or standing and are likely to identify with an organization that holds high prestige. Since employees tend to have prosocial values and expect those values to be implemented by their organization, CSR initiatives may raise employees’ attachment and identification to the organization (Wang et al., 2019). As a result, such a feeling of organizational identification may motivate employees to perform more positively, which in turn improves their job performance. Aligned with SIT, we propose that CSR initiatives will be positively related to employees’ job performance.

**Hypothesis 1:** CSR initiatives will have a positive association with employees’ job performance.

### The Mediating Effect of Industrial Relations Climate

Given the theoretical framework and empirical evidence presented by prior research, we propose that IRC mediates the link between CSR initiatives and job performance. According to SIPT, the environment where employees spend their time affects their attitudes toward an organization by directly providing attitudes, behavioral guidance, transferable beliefs, and needs (Salancik and Pfeffer, 1978). Employees are aware of their work environment, and the consequences of certain behaviors largely determine their actions. The theoretical background has been advanced by human resource management and organizational behavior literature, which suggests that an organization’s CSR practices are significant resources that improve organizational culture and subsequent employees’ attitudes and behaviors (Mossholder et al., 2011). As one of the most important organizational strategies, CSR initiatives care about both external and internal stakeholders as well as a culture that respects sustainability, in addition to profit maximization. CSR initiatives integrate the value of sustainability into organizational systems and motivate employees to react similarly in daily life (Schneider et al., 2013; Shen and Zhang, 2019). As such, the organization most probably develops an organizational IRC, which is the shared perception about the work environment targeted at addressing the mutual relations between the organization and various stakeholders.

In addition, numerous studies have proposed empirical evidence for the mediating role of organizational climates in organizational policies. Shedding light on the input–transformation–output mechanism, Masters et al. (2006) presented insights on an organization’s management practices, which can affect employees’ attitudes, behaviors, and organizational outcomes. Xi et al. (2017) also suggested that partnership practices could positively affect organizational IRC and, in turn, improve employees’ attitudes. Based on these findings, IRC establishes a link between CSR initiatives and employees’ job performance, indicating that IRC plays a mediating role in the association between CSR initiatives and job performance. Thus, we postulate:

**Hypothesis 2:** IRC mediates the relationship between CSR initiatives and job performance.

### The Mediating Role of Psychological Contract Fulfillment

Compared with explicit terms and requirements in a written form, the psychological contract provides ideas with respect to employee perceptions and beliefs about their implicit obligations (Rousseau, 2001). It highlights the cognitive perception of mutual obligations established between employees and organizations (Robinson and Rousseau, 1994). The psychological contract theory is deeply grounded in SET (Blau, 1964) and reciprocity (Gouldner, 1960). Employees’ affinity to reciprocate the organization’s values depends on whether the organization fulfills their needs and expectations (Farnese et al., 2018). However, when employees perceive a mismatched relationship between their work effort and acceptable resources as well as inducements, the exchange is likely to be considered an unbalanced state. Consequently, employees may get an impression that the psychological contract is not being honored (Birtch et al., 2016).

Based on SET, a wide variety of researchers have examined the antecedents and outcomes of PCF. For example, some predictors could create a favorable environment that can meet employees’ expectations, which boosts the relationship between employees and the organization. Organizational factors, including HR practices (Latorre et al., 2016, 2020), leadership (Jiang et al., 2017), and organizational culture (Richard et al., 2009), have been recognized as key antecedents of the psychological contract. In addition, employees’ attitudes and behavior outcomes have been investigated, including organizational commitment (Jabeen et al., 2015), job satisfaction (Raja et al., 2011), turnover intention (Chen and Wu, 2017), organizational citizenship behavior (Shih and Chen, 2011), and job performance (Cheung et al., 2017). In the current study, we seek to explore the mediating effect of PCF on the relationship between CSR initiatives and job performance. According to previous evidence, successful exchanges would be shaped as a consequence of organizations adopting organizational-level CSR initiatives (e.g., providing employees’ needs and promoting the well-being of society), as well as care and concern for its stakeholders (Kutaula et al., 2020). When the fulfillment of organizational responsibilities and obligations raises employees’ expectations, employees would demonstrate loyalty and commitment to the organization and, subsequently, improve their job performance. Thus, we argue that:

**Hypothesis 3:** Psychological contract fulfillment mediates the relationship between CSR initiatives and job performance.

## Research Methods

### Sample and Procedure

Given the nature of our study, the samples in this study were collected based on companies that had CSR strategies listed in the local government system. To recruit the participants, we first contacted these companies by telephone and explained the process of our study. After showing the purpose of the current study, 85 companies (89.47%) from different sectors agreed to participate in our research. Before providing the questionnaires, we sent cover letters, including detailed instructions to each company’s human resources (HR) department. The HR director was asked to select employees randomly to avoid response bias. To minimize common method bias (Podsakoff et al., 2012), our data were collected from multiple sources, including middle managers, HR directors, frontline employees, and their direct superiors. The first part of the questionnaire was for HR directors to provide basic information about the company, including its industry, size, and ownership. Although top managers have been regarded as designers of CSR strategy within organizations, middle managers play a critical role in enforcing top-down vision and implementing CSR strategies in practice (Maon et al., 2009). Thus, middle managers were asked to access the CSR initiatives of their companies. The data on IRC and PCF were collected from employees, and their direct supervisors were responsible for evaluating employees’ job performance. After removing invalid questionnaires, we were left with valid responses from 764 supervisor–subordinate dyads and 271 middle managers who had assessed the questionnaires. The response rates were thus 93.63% for the employees and 92.90% for the companies.

Participating companies ranged in size of employees from 100 to 13,000; 6 of the 79 firms were in the agricultural industry, 18 were in the manufacturing industry, and 55 were in the service industry. Among those companies, 44.4% were state-owned enterprises, 33.2% were private-owned enterprises, and 17.3% were foreign-owned enterprises.

Of the 764 employees sampled, gender-wise, 51.2% were male; education-wise, 7.6% were high school and below, 20.8% were junior college, 67.0% graduated from university, and 4.6% had a master’s degree; age-wise, 12.2% were 18–25 years old, 53.8% were aged 26–35 years, 31.1% were aged 36–45, and 2.9% were aged 46–55 years; and tenure-wise, 88.2% had worked for more than 1 year (Table 1).

**TABLE 1:** Data sample characteristics of employees.

		Frequency	Percent (%)
Gender	Male	391	51.2
	Female	373	48.8
Age	18–25	93	12.6
	26–35	411	53.8
	36–45	238	31.1
	46–55	22	2.9
Education	High school and below	58	7.6
	Junior college	159	20.8
	Undergraduate	512	67.0
	Master’s degree	35	4.6
Tenure	Less than 1 year	90	11.8
	1–5 years	214	28.0
	6–10 years	226	29.6
	more than 10 years	234	30.6

**N = 764.**

### Measures

We used a five-point Likert scale ranging from 1 (*strongly disagree*) to 5 (*strongly agree*) to measure all variables in this study. Since the scales were originally developed in English, a back-translated method was adopted to ensure the accuracy of the meaning (Brislin, 1980). We invited two English–Chinese bilinguals to undertake the translation of measurements in our study. Bilingual 1 translated English measurements into Chinese and then bilingual 2 translated the newly translated version back into English. After the English–Chinese and Chinese–English translation processes, one researcher compared the original items with the newly translated version. The final pretesting step was undertaken with three HR experts to ensure the relevance and validity of the measurements. After confirming the original language and back-translated versions without any perceived differences, the questionnaires were distributed to the employees and their supervisors.

#### CSR Initiatives

We used the 17-items scale adopted from Turker (2009) to measure CSR initiatives. Sample items for each subdimension are as follows: “Our company makes investment to create a better life for future generations,” “The management of our company is primarily concerned with employees’ needs and wants,” “Our company provides full and accurate information about its products to its customers,” and “Our company complies with legal regulations completely and promptly.” The Cronbach’s alpha of each subdimension was 0.90, 0.86, 0.87, and 0.83, respectively. Following the suggestions by Hur et al. (2018), we employed an overall construct to measure organizational CSR. The confirmatory factor analyses showed an acceptable fit for the one-factor structure: χ^2^/*df* = 1.18, GFI = 0.95, comparative fit index (CFI)° = 0.99, root mean square error of approximation (RMSEA) = 0.03, and standardized root mean square residual (SRMR) = 0.04. The Cronbach’s alpha for the overall CSR scale was 0.90.

#### Industrial Relations Climate

IRC was measured using a six-item scale developed by Hammer et al. (1991). The scale was subsequently validated by Snape and Redman (2012). A sample item is “Workers and management distrust one another (R).” The Cronbach’s alpha for the overall IRC scale was 0.94.

#### Psychological Contract Fulfillment

We measured PCF using a 14-item scale from Shih and Chen (2011). A sample item is “This company is responsive to employee concerns and well-being.” Cronbach’s alpha for PCF was 0.88.

#### Job Performance

We measured job performance using a 19-item scale from Van Scotter and Motowidlo (1996). A sample item for this scale is “He/She takes the initiative to solve a work problem.” The Cronbach’s alpha for job performance was 0.94.

#### Control Variables

Prior research has indicated that employees’ demographic variables are associated with job performance (Newman et al., 2015). Thus, we control for employee gender, education, and age, as well as organizational tenure, and tested whether job performance could be influenced by these demographic variables.

### Analytical Strategy

Given the nested structure of the data, we calculated intraclass correlation coefficients (ICCs) and interrater agreement values (Rwg) to assess the validity of aggregating data for CSR initiatives and industrial relations climate. Reliability of score within group ICC(1) estimates the proportion of the total variance of a measure that is explained by unit membership, while the reliability of mean group score ICC(2) measures the reliability of the group mean scores (Bliese, 2000). Rwg represents the within-group agreement for the given measure. The results of intraclass correlation coefficients [ICC(1) = 0.35, ICC(2) = 0.86] as well as the interrater agreement value [Rwg = 0.77] supported the aggregation of the employees’ individual responses to the organizational-level IRC (Bliese, 2000). Moreover, we tested the aggregation statistics for CSR initiatives that were answered by the middle manager (*n* = 271), supporting an aggregation to the firm level [ICC(1) = 0.43, ICC(2) = 0.80, Rwg = 0.96]. The in-between parts of the model were designed to test Hypotheses 1, 2, and 3, including the direct and indirect effects of organizational CSR initiatives on employees’ job performance through IRC and PCF. Since the collected data were nested within organizations, we employed multilevel structural equation modeling (MSEM) to examine the proposed hypotheses by using Mplus 7.4 software (Muthén and Muthén, 1998–2018). According to previous studies, MSEM can overcome the limitations of traditional multilevel mediation analysis. Moreover, we adopted the Monte Carlo method to assess the mediating effect *via* 95% confidence interval results (Preacher et al., 2010).

## Results

### Confirmatory Factor Analyses

To test the distinctiveness among three individual-level variables, including industrial relations climate, psychological PCF, and job performance, we conducted a series of confirmatory factor analyses. The three-factor model showed a good model fit, χ^2^ = 1,025.72, *df* = 693, CFI = 0.98, Tucker–Lewis index (TLI) = 0.98, SRMR = 0.03, RMSEA = 0.03. Compared with the alternative models, the three-factor model indicated a better model fit. Furthermore, we employed multilevel confirmatory factor analysis to estimate the model fit of the hypothesized model (χ^2^ = 2,205.15, *df* = 1,958, CFI = 0.99, TLI = 0.99, SRMR = 0.03, RMSEA = 0.01). The results suggested that the factor structure developed in our model was acceptable at between-group levels of analysis (Table 2).

**TABLE 2:** Results of confirmatory factor analysis.

	χ^2^	*df*	CFI	TLI	SRMR	RMSEA
Three factors	1025.72	693	0.98	0.98	0.03	0.03
Two factors (IRC + JP) + PCF	4277.95	699	0.80	0.79	0.08	0.08
Two factors (IRC + PCF) + JP	5102.24	699	0.75	0.74	0.10	0.09
One factor (IRC + PCF + JP)	8666.81	702	0.55	0.53	0.13	0.12

**IRC, industrial relations climate; PCF, psychological contract fulfilment; JP, job performance.**

### Descriptive Statistics

Table 3 shows the descriptive statistics, including the means, standard deviations, and correlations for the individual- and organizational-level variables. Middle managers’ perceptions of CSR initiatives were positively related to the IRC (*r* = 0.36, *p* < 0.01), PCF (*r* = 0.27, *p* < 0.01), and job performance (*r* = 0.55, *p* < 0.01). Moreover, the IRC (*r* = 0.27, *p* < 0.01) and PCF (*r* = 0.49, *p* < 0.01) were all positively related to employees’ job performance.

**TABLE 3:** Means, standard deviation, and correlations among variables.

	Mean	SD	1	2	3	4	5	6	7	8
***Individual level***										
1. Gender	0.51	0.50								
2. Age	2.25	0.70	0.02							
3. Education	2.69	0.68	0.00	0.12**						
4. Tenure	2.79	1.01	–0.04	0.32**	0.19**					
5. PCF	2.97	0.64	0.05	0.02	–0.01	–0.01	(0.88)			
6. JP	3.19	0.86	0.02	0.03	0.03	0.02	0.49**	(0.94)		
***Firm level***										
7. CSR initiatives	2.82	0.57	–0.03	0.04	–0.02	0.03	0.27**	0.55**	(0.90)	
8. IRC	3.17	0.89	0.05	0.02	0.02	0.02	0.07	0.27**	0.36**	(0.94)

**p < 0.01. Reliability coefficients were listed on the diagonal.

### Hypotheses Testing

Following the suggestions by Boehm et al. (2014), we investigated our assumed hypotheses using MSEM. The overall results of the proposed model indicated a good fit (χ^2^ = 1,409.78, *df* = 1,055, CFI = 0.97, TLI = 0.97, SRMR = 0.03, RMSEA = 0.02). Hypothesis 1 proposed that CSR initiatives would have a positive effect on job performance. As shown in Table 4, CSR initiatives were significantly related to employees’ job performance (β = 0.57, *p* < 0.001). Hence, Hypothesis 1 was supported.

**TABLE 4:** Results of cross-level indirect effects.

Indirect effect	Indirect effect	Confidence interval
***Cross-level indirect effect***		
CSR →industrial relations climate → job performance	0.12**	[0.048, 0.205]
CSR→ psychological contract→ job performance	0.07*	[0.019, 0.140]

*p < 0.05, **p < 0.01.

Hypothesis 2 postulated that industrial relations climate mediates the relationship between CSR initiatives and job performance. The results revealed that industrial relations climate played a mediating role in the relationship between CSR initiatives and job performance, with an indirect effect of 0.12 (95% CI = [0.048, 0.205], *p* < 0.01). Thus, Hypothesis 2 was supported.

Hypothesis 3 predicted that PCF mediates the relationship between CSR initiatives and job performance. The results showed that PCF played a mediating role in the relationship between CSR initiatives and job performance, with an indirect effect of 0.07 (95% CI = [0.019, 0.140], *p* < 0.05). Thus, Hypothesis 3 was also supported.

## Discussion

During the last two decades, scholars have called for investigating the microfoundation of CSR research and exploring employees’ responses to CSR (Rupp et al., 2013; Wang et al., 2017). Our study was conducted to examine the direct effect of CSR initiatives on employees’ job performance. In addition, we explored the mediating role of IRC and PCF in the relationship between CSR initiatives on job performance by considering SET and SIPT (Blau, 1964; Salancik and Pfeffer, 1978). Our results support the hypothesized relationships. The results show that, in addition to the direct impact of CSR initiatives on job performance, CSR initiatives also have an indirect effect *via* IRC and PCF. This study presents several theoretical and practical implications.

### Theoretical Implications

This research enriches the CSR literature in four notable approaches. First, the results revealed that CSR initiatives were positively related to employees’ job performance. Although several studies have examined the role of employees’ perceptions of CSR in predicting job attitudes and job behaviors (Newman et al., 2019), this study indicates that CSR activities that benefit social and non-social stakeholders can also improve employees’ positive outcomes. The current study verified prior studies by supporting the hypothesis that CSR initiatives would have a positive effect on employee work behavior (e.g., Wang et al., 2019). The results of this study suggest that when an organization implements CSR activities, employees may feel a sense of belonging and identification with the organization. Since CSR is a critical source of employees’ pride, they may feel that they should achieve higher performance in organizations with a good societal reputation. Therefore, this study contributes to SIT by identifying the CSR–job performance relationship.

Second, the results support the idea put forth by Glavas (2016), who proposed that an integrated image of CSR should be built by incorporating analysis at the individual level. Prior CSR research is mostly conducted at the macro and institutional levels, which focus on the impact on organizational performance (Frynas and Yamahaki, 2016; Serra-Cantallops et al., 2018). However, our findings suggest that CSR initiatives are interpreted as the organization’s social responsibility activities that could improve employees’ behavioral outcomes. Thus, our study enriches the CSR literature by interpreting CSR as an organizational-level predictor of job performance.

Third, this study contributes to existing research by examining dual mediating mechanisms in the relationship between CSR initiatives and job performance. By confirming the mediating effect of IRC and PCF in CSR–job performance relationships, the current study responds to the call to explore stakeholder relations mediators (Aguinis and Glavas, 2012). The findings of this study show that CSR initiatives are considered significant signals of organizations’ sustainable practices and are likely to promote harmonious organization–employee relationships. These are identified as centrally important issues in building a long-term employment relationship that is likely to reciprocally improve between organizations and employees (Guest, 2004; Latorre et al., 2016). Consequently, both IRC and PCF are conducive to improving employees’ productivity. To the best of our knowledge, this is the first study to explore the relationship between CSR and job performance, accounting for dual mediators of IRC and PCF. Moreover, our study bridged multilevel gaps, thereby unveiling the black box of the transmitting processes right from CSR initiatives to job performance. Therefore, these findings shed light on establishing an integrated framework about why and how organizational CSR initiatives affect employees’ behavior.

Finally, although IRC is a crucial organizational factor that could predict employees’ outcomes (e.g., Pyman et al., 2010; Valizade et al., 2016; Fortin-Bergeron et al., 2018), in the existing IRC literature, there are few studies with a non-Western context. Mirroring China’s rapid growth, the Chinese government has implemented several reforms to build harmonious labor relations (Newman et al., 2019). However, labor relations problems in Chinese enterprises have steadily increased (Xi et al., 2017), which affects the performance and development of enterprises. It is worth further exploring whether the research results of IRC based on the Western cultural context are applicable to Chinese contexts. Thus, the current study also provides an avenue for researchers to compare a series of IRC practices from the cross-cultural perspective and helps to build more universally acceptable theories.

### Managerial Implications

The findings of the current study also provide some practical implications. First, although there are inconsistent scholarly conclusions relating CSR’s benefits to increasing additional costs, our study demonstrates that CSR initiatives as are a win-win strategy that could improve employee’s job performance. Thus, middle managers should be aware that an organization’s actual CSR initiatives may influence employees’ behaviors, which in turn improve organizational competitiveness. In order to increase employees’ job performance, organizations should allocate more resources to implementing CSR initiatives. In addition, we suggest that organizations should implement CSR initiatives encompassing social and non-social dimensions that reflect the company’s management philosophy and, thus, implement CSR initiatives into daily operations and management activities.

Second, the results suggest that CSR initiatives may also affect the principal internal stakeholders (McWilliams and Siegel, 2001). To facilitate better job performance, organizations should communicate regularly to employees about CSR initiatives and the ways in which these sustainable practices affect society. We suggest that organizations should focus on promoting internal CSR initiatives. Employees are the major ones who benefit from internal CSR initiatives, and in turn, they will be obliged to respond positively to the organization (Bhattacharya et al., 2009; De Roeck et al., 2014). Thus, organizations should implement more CSR initiatives to employees, such as developing a fair reward system for employees, providing them learning and training opportunities.

Third, given the mediating role of the results, positive CSR influences job performance *via* IRC and PCF. Since the critical role of workplace partnership is creating positive industrial relations, organizations should consider how CSR initiatives are experienced by employees and their degree of participation in management activities. Additionally, as psychological contracts are framed in accordance with CSR communications for employees who are perceived as internal stakeholders (Maignan and Ferrell, 2004), organizations are recommended to reinforce communications with CSR promises to social and non-social stakeholders and to proactively seek feedback from employees thereon (Kiazad et al., 2019).

### Limitations and Future Research

This study has a few limitations that should be considered in future research. First, this study uses multilevel sources to verify the relationship between various variables, which can effectively overcome the common method bias issue (Podsakoff et al., 2012). However, due to the limitation of cross-sectional data, causal inference on the relationship could not be established in this study. Thus, we suggest that future studies could employ a longitudinal design to offer meaningful evidence to support the findings of our study. Second, this study adopted a multilevel approach that introduced dual mediators, namely IRC and PCF, to examine the positive consequences of CSR initiatives. However, antecedents of CSR initiatives have been neglected in our hypothesized model. Scholars could consider the combination of multitheory (e.g., stakeholder theory and legitimacy theory) to illustrate how social and environmental resources affect CSR activities (Frynas and Yamahaki, 2016). Third, although the findings of this study indicate that CSR initiatives could improve job performance, prior studies have stressed the boundary condition that may influence employees’ CSR judgments that subsequently affect the effectiveness of CSR initiatives. Therefore, other moderating variables, such as managers’ directive leadership, should be introduced to our model and explain when and how CSR initiatives could successfully lead to employees’ attitudes and behavioral outcomes.

## Data Availability Statement

The raw data supporting the conclusions of this article will be made available by the authors, without undue reservation.

## Ethics Statement

Ethical approval was not provided for this study on human participants because ethical review and approval was not required for the study on human participants in accordance with the local legislation and institutional requirements. Written informed consent from the participants was not required to participate in this study in accordance with the national legislation and the institutional requirements. Written informed consent for participation was not required for this study in accordance with the national legislation and the institutional requirements. Written informed consent was not obtained from the individual(s) for the publication of any potentially identifiable images or data included in this article.

## Author Contributions

MYF designed the study, wrote the manuscript, and collected the data. PF wrote the manuscript and analyzed the data. SN and P-CC reviewed and revised the manuscript. All authors contributed to the article and approved the submitted version.

## Conflict of Interest

The authors declare that the research was conducted in the absence of any commercial or financial relationships that could be construed as a potential conflict of interest.
